# Factors Associated With Self-Rated Health Among Older Adults in Japan: A Decision Tree Analysis

**DOI:** 10.7759/cureus.84245

**Published:** 2025-05-16

**Authors:** Hirotomo Shibahashi, Kanta Ohno, Yosuke Seike, Shinpei Ikeda

**Affiliations:** 1 Occupational Therapy, Department of Rehabilitation, School of Health Sciences, Tokyo University of Technology, Tokyo, JPN; 2 Faculty of Social Welfare, University of Kochi, Kochi, JPN; 3 Division of Occupational Therapy, Department of Rehabilitation, Faculty of Medical Sciences, Shonan University of Medical Sciences, Yokohama, JPN

**Keywords:** decision tree analysis, depressive symptoms, japanese older adults, physical activity, self-rated health

## Abstract

Background

Self-rated health (SRH) is a widely used single-item measure that predicts morbidity, mortality, and healthcare use. In aging societies, such as Japan, SRH serves as a vital public health indicator. Although many factors influence SRH, their relative importance and interactions remain unclear, particularly among older adults. Prior studies have mostly used linear models, which are limited in their ability to capture interactions and non-linear relationships. Such complexities are often present in multifactorial outcomes such as SRH. This study aimed to identify the key determinants of SRH using decision tree analysis in a large sample of community-dwelling older adults in Japan to inform targeted strategies for promoting healthy aging.

Method

We analyzed cross-sectional data from 1,821 older adults in Ayase City, Japan, corresponding to a response rate of 62.1% from 3,058 individuals invited by mail. SRH was dichotomized into high and low categories. Missing data were addressed using multiple imputations. Decision tree analysis using the classification and regression tree (CART) algorithm identified the key determinants of SRH, focusing on modifiable factors. The predictors included age, sex, Geriatric Depression Scale (GDS) score, Motor Fitness Scale (MFS) score, instrumental activities of daily living (IADL) assessed by the Tokyo Metropolitan Institute of Gerontology Index of Competence (TMIG-IC), and the frequency of going out and exercising. The model performance was evaluated using 10-fold cross-validation.

Results

Among the 1,821 older adults, 73.5% were classified as belonging to the high SRH group. Higher MFS scores, lower GDS scores, greater TMIG-IC scores, and more frequent going out and exercise were significantly associated with a high SRH (all p < 0.001). Decision tree analysis identified MFS as the most important discriminator, followed by GDS and activity frequency. The model achieved an accuracy of 80.3%, with a specificity of 90.8% and a sensitivity of 51.5%.

Conclusions

Using decision tree analysis, this study identified MFS, GDS, and TMIG-IC as key determinants of SRH among older adults in Japan. These modifiable factors, including physical function, mental health, and daily competence, offer actionable targets for health promotion. The model’s ability to stratify SRH based on practical variables supports its use in guiding individualized and population-level strategies. These findings highlight the importance of addressing motor fitness, depressive symptoms, and functional autonomy through community-based exercise programs, mental health screening, and IADL-enhancing services, in order to improve perceived health and quality of life in aging populations. However, due to its modest sensitivity, the model may be less effective in detecting individuals with low SRH and should be used alongside other screening tools when applied in population health settings.

## Introduction

Self-rated health (SRH) is a widely used single-item measure that reflects an individual’s overall perception of their physical and mental well-being. Despite its simplicity, SRH has consistently demonstrated strong predictive validity for morbidity, mortality, and healthcare utilization in diverse populations [1-3]. In the context of rapidly aging societies, such as Japan, where the proportion of older adults continues to rise, SRH has become a particularly important indicator for public health monitoring and the evaluation of health interventions [4,5]. Identifying factors that influence SRH among older adults may contribute not only to improving individual quality of life but also to reducing future healthcare burdens on society [3,5].

Previous studies have identified various determinants associated with SRH in older populations. These include physical factors such as functional ability and the presence of chronic diseases, mental health indicators such as depressive symptoms, and social determinants such as social support, the frequency of social participation, and socioeconomic status [5,6]. However, traditional regression-based approaches are often constrained by assumptions of linearity and additivity among predictors. These limitations may hinder their ability to capture complex interactions and non-linear relationships that are frequently present in multifactorial health outcomes such as SRH. In contrast, decision tree models such as the classification and regression tree (CART) provide a flexible and nonparametric framework that facilitates the identification of hierarchical structures and interaction effects. This enhances both the interpretability and practical applicability of the findings in real-world settings [7]. In Japan, several community-based studies have reported associations between higher SRH and greater physical activity, better nutritional status, and lower psychological distress [8]. However, most of these findings have been based on traditional multivariate regression models, which, although informative, are limited in their ability to detect complex non-linear relationships or interactions between variables. This limitation is particularly important in the context of SRH, as subjective health perceptions among older adults are often shaped by complex interactions between physical, mental, and social factors. For example, the impact of physical activity on SRH may differ depending on an individual’s level of psychological distress or functional autonomy. By capturing such interaction effects, decision tree analysis provides a more nuanced understanding of how multiple factors jointly influence perceived health.

While many correlates of SRH have been identified, the relative importance and hierarchical structure of these factors remain unclear, particularly in the Japanese older adult population. Given the wide range of physical, psychological, and social variables that may influence SRH, it is essential to clarify how these factors interact and which factors play the most dominant roles. While some studies have utilized data-driven approaches, such as decision tree analysis, to explore the determinants of SRH, these remain limited in number and have not been applied to older adults in Japan [9-11]. In this study, we focused on modifiable factors to enhance the practical utility of the findings and therefore excluded non-modifiable variables such as income, education, and physician-diagnosed chronic conditions from our analysis.

This study aimed to identify the key determinants of SRH among community-dwelling older adults in Ayase City, Japan, using decision tree analysis. Ayase City is a mid-sized suburban municipality in the Tokyo metropolitan area, with a demographic profile and aging rate comparable to the national average. Its characteristics are broadly similar to many other semi-urban cities in Japan, making it a practical setting for community-based aging research. By leveraging a large-scale municipal dataset that included physical, mental, and lifestyle-related variables, we aimed to uncover the hierarchical structure of the factors most strongly associated with SRH. Through this approach, we sought to provide new insights that could inform targeted public health strategies for promoting healthy aging in local Japanese populations. In this study, “healthy aging” is defined, following the World Health Organization, as the process of developing and maintaining functional ability that enables well-being in older age. The term “older adults” refers to community-dwelling individuals aged 65 years or older, whose health status may range from functionally independent to mildly impaired.

## Materials and methods

Study participants

This cross-sectional study analyzed secondary data derived from the “Survey on Health and Life of Older Adults,” targeting community-dwelling individuals aged 65 years or older residing in Ayase City, Kanagawa Prefecture, Japan. The survey was administered via postal mail between June 28 and July 9, 2017. Of the 3,058 individuals invited to participate, 1,899 returned the completed questionnaires. After excluding respondents with missing data on key variables required for the main analysis (as described below) and those with extensive item nonresponse that precluded reliable interpretation, a final analytic sample of 1,821 community-dwelling older adults was retained. The study protocol was reviewed and approved by the Ethics Committee of J. F. Oberlin University (approval number: 17007). The completion and return of the questionnaire were considered to constitute informed consent, as approved by the ethics committee.

Measurements

Demographic and health-related data collected in the survey included age, sex, years of education, SRH, physician-diagnosed chronic conditions, depressive symptoms assessed using the Geriatric Depression Scale (GDS), motor performance measured using the Motor Fitness Scale (MFS), and frequencies of going out and engaging in exercise. Educational attainment was categorized into four groups based on cumulative years of schooling since elementary education: ≤6 years, 7-9 years, 10-12 years, and ≥13 years.

SRH was assessed using a four-point Likert scale with response options: “very healthy,” “somewhat healthy,” “not very healthy,” and “unhealthy.” Respondents also indicated whether they had been diagnosed by a physician with any of the 24 specified chronic conditions, including hypertension, cerebrovascular disease, osteoporosis, and diabetes.

Depressive symptoms were evaluated using a short-form version of the GDS, a self-administered tool developed to minimize the cognitive burden among older adults. The five-item scale includes questions on life satisfaction, boredom, preference for staying at home, sense of purpose, and feelings of helplessness. Responses were binary, with total scores ranging from 0 to 5, with higher scores indicating more severe depressive symptoms [12].

Instrumental activities of daily living (IADL) were assessed using items from the Tokyo Metropolitan Institute of Gerontology Index of Competence (TMIG-IC). The TMIG-IC consists of 13 items that evaluate functional independence in instrumental self-maintenance, intellectual activities, and social roles. For this study, five items across these domains were selected, and the scores were summed to indicate higher IADL functioning [13]. The five TMIG-IC items used in this study assessed instrumental activities of daily living (IADL) and included the following: (1) going out alone using public transportation such as buses or trains, (2) managing personal finances including deposits and withdrawals, (3) visiting friends at their homes, (4) offering advice or emotional support to family members or friends, and (5) shopping for daily necessities.

Motor fitness was measured using the MFS, which includes 14 binary items that assess mobility, muscular strength, and balance. Scores range from 0 to 14, with higher values representing better physical performance. The frequency of going out and exercise participation was evaluated on a five-point scale ranging from “less than once a week” to “almost every day” [14].

Statistical analysis

Missing data were handled using multiple imputation by chained equations (MICE) with predictive mean matching (PMM), following Rubin’s framework under the assumption of missing data at random (MAR) [15]. To ensure convergence and improve estimation stability, 20 imputed datasets were created using the multiple imputation by chained equations (MICE) method, with 50 iterations per dataset. Predictive mean matching (PMM) was used as the imputation method, and a fixed random seed (1234) was applied to ensure reproducibility. This approach replaces missing values with plausible estimates derived from observed data, thereby minimizing potential bias due to nonresponses. Given that the overall proportion of missing data exceeded 10%, multiple imputations were adopted to enhance the robustness of the parameter estimation. Missingness was most prevalent in the MFS (12.4%), followed by the GDS (6.3%) and self-reported frequency of exercise (5.7%). In contrast, demographic variables such as age and gender had minimal or no missing values. The imputation model incorporated all analytic variables to ensure internal consistency and preserve associations between them. Increasing the number of imputations is particularly beneficial when the fraction of missing information is substantial, as it reduces the variability in confidence intervals (CI) and p-values [16].

The participants were divided into two groups based on their SRH responses: individuals who responded “very healthy” or “somewhat healthy” were classified into the high SRH group, while those who responded “not very healthy” or “unhealthy” were classified into the low SRH group. Differences in continuous variables between the groups were evaluated using the Mann-Whitney U test. For categorical variables, chi-square tests were applied, and when evaluating physician-diagnosed conditions with a total prevalence of ≥5% across both SRH groups, the G test was employed to address distributional skew.

To identify the factors associated with SRH, decision tree analysis was conducted using the CART algorithm. SRH responses were dichotomized into high (combining “very healthy” and “somewhat healthy”) and low (combining “not very healthy” and “unhealthy”) categories for analytic purposes. The CART method was selected for its ability to model complex, non-linear relationships and automatically detect interaction effects without the need for a priori specification of variable interactions. To enhance the interpretability and actionability of the findings, we intentionally excluded non-modifiable factors, such as education and physician-diagnosed chronic diseases, from the model. This allowed the analysis to focus on variables that could be directly influenced by public health or clinical interventions.

All candidate variables, including age, sex, GDS, TMIG-IC, MFS, the frequency of going out, and the frequency of exercise, were entered into the decision tree model to examine the factors associated with SRH. These variables were selected based on prior research demonstrating their relevance to SRH and the quality of life in older adults, particularly in relation to physical function, psychological status, and lifestyle behaviors [5,6]. The Gini impurity index was used as the splitting criterion in this study. Tenfold cross-validation was performed to assess the robustness of the model and prevent overfitting. The relative importance of the predictor variables was calculated to determine their contribution to the classification accuracy.

All statistical analyses were conducted using the R software (version 4.2.1; R Foundation for Statistical Computing, Vienna, Austria) with the following packages: haven, labelled, tidyverse (including dplyr and ggplot2), mice, rpart, rpart.plot, caret, and rattle. Conventional statistical tests were considered significant at a two-tailed p-value of <0.05. Model validity for the decision tree analysis was assessed based on the misclassification rate and the cross-validated classification error. The decision tree model was implemented using the rpart package in R. The complexity parameter (CP) was set to 0.01 to control overfitting. Other parameters were left at default settings, including minsplit = 20, maxdepth = 30, and minbucket = 7.

## Results

Descriptive statistics

A total of 1,821 community-dwelling older adults were included in the final analysis, of whom 1,337 (73.5%) were classified into the high SRH perception group and 484 (26.6%) into the low SRH perception group. The median age was significantly lower in the high SRH group than in the low SRH group (74 years {interquartile range (IQR): 70-78} versus 77 years {IQR: 72-81}, p < 0.001). The proportion of female participants was similar between the groups (54.3% versus 53.7%, p = 0.832). Although sex is often considered a relevant factor in SRH, findings across studies have been inconsistent. This comparison was included to explore potential sex-related differences, but no significant association was observed in this sample.

Educational attainment differed significantly between the groups (p < 0.001), with the high SRH group having a higher proportion of individuals with ≥13 years of education (34.2% versus 24.0%) and a lower proportion of those with ≤9 years of education (16.2% versus 27.5%).

The number of diagnosed chronic conditions was significantly associated with the SRH group status (p < 0.001). Most individuals in the high SRH group had 0-2 diagnosed conditions (87.7%), whereas the low SRH group had a greater proportion of individuals with three or more conditions (43.8%).

Significant between-group differences were observed for several physician-diagnosed diseases. Significant associations were also identified between SRH and physician-diagnosed conditions (p < 0.001) (Table 1). Among these, hypertension exhibited the largest between-group difference in prevalence (31.1% in the low SRH group versus 18.3% in the high SRH group), suggesting a potential association with lower subjective health perception. The full list of physician-diagnosed conditions and their distributions by SRH perception group are presented in Table 2. In Table 1, only conditions with a total prevalence of ≥5% across both groups are displayed to enhance clarity and comparability.

**Table 1:** Participant characteristics grouped by SRH †Mann-Whitney U test (U value shown) ‡Chi-square test (χ² value shown) §G test (G value shown) SRH, self-rated health; IQR, interquartile range

Variable	High SRH perception group (n = 1,337)	Low SRH perception group (n = 484)	Test statistic	P-value
Age (median, IQR)	74 (70-78)	77 (72-81)	U = 253,247	<0.001^†^
Sex (%)				
Female	726 (54.3)	260 (53.7)	χ^2^ = 0.027831	0.832^‡^
Male	611 (45.7)	224 (46.3)		
Years of education (%)				
6 years or less	6 (0.4)	12 (2.5)	χ^2^ = 45.905	<0.001^‡^
7-9 years	211 (15.8)	121 (25.0)		
10-12 years	622 (46.5)	213 (44.0)		
13 years or more	457 (34.2)	116 (24.0)		
No response	41 (3.1)	22 (4.5)		
Number of diagnosed conditions (%)				
0-2	1,172 (87.7)	272 (56.2)	χ^2^ = 218.87	<0.001^‡^
3-5	159 (11.9)	194 (40.1)		
6-8	6 (0.4)	18 (3.7)		
Diagnosed conditions by physician (%)				
Hypertension	503 (31.1)	217 (18.3)	G = 98.644	<0.001^§^
Osteoporosis	63 (3.9)	76 (6.4)		
Spinal canal stenosis	47 (2.9)	54 (4.6)		
Osteoarthritis	84 (5.2)	63 (5.3)		
Cataract	102 (6.3)	87 (7.4)		
Glaucoma	65 (4.0)	37 (3.1)		
Hearing loss	64 (4.0)	55 (4.6)		
Diabetes mellitus	137 (8.5)	96 (8.1)		
Hyperlipidemia	154 (9.5)	72 (6.1)		
Benign prostatic hyperplasia (BPH)	65 (4.0)	43 (3.6)		
Cancer	27 (1.7)	66 (5.6)		

**Table 2:** Distribution of physician-diagnosed conditions by SRH perception group (multiple responses allowed) SRH: self-rated health

Variable	High SRH perception group (n = 1,337)	Low SRH perception group (n = 484)
Hypertension	503	217
Stroke (including cerebral hemorrhage and cerebral infarction)	23	46
Osteoporosis	63	76
Rheumatoid arthritis	19	23
Spinal canal stenosis	47	54
Osteoarthritis	84	63
Fracture	9	17
Cataract	102	87
Glaucoma	65	37
Hearing loss	64	55
Diabetes mellitus	137	96
Hyperlipidemia	154	72
Angina pectoris	31	36
Myocardial infarction	16	15
Bronchial asthma	35	17
Pneumonia	4	12
Chronic obstructive pulmonary disease (COPD)	5	3
Renal failure	7	22
Benign prostatic hyperplasia (BPH)	65	43
Gastric or duodenal ulcer	22	17
Cancer	27	66
Dementia	14	25
Parkinson’s disease	6	11
Others	116	73

Table 3 presents a comparative analysis of the GDS, TMIG-IC, and MFS scores and activity frequency between older adults with high and low SRH. Compared with those with low SRH, the participants in the high SRH group exhibited significantly lower GDS scores (median, 0 versus 2; p < 0.001; r = -0.49; and 95% CI, -0.53, -0.44) and TMIG-IC scores (median, 5 versus 4; p < 0.001; r = 0.32; and 95% CI, 0.26, 0.37). They also demonstrated a higher overall MFS score (median, 13 versus 9; p < 0.001; r = 0.59; and 95% CI, 0.54, 0.63), including significantly greater mobility, strength, and balance subdomain scores. Moreover, the high SRH group reported more frequent engagement in both going out and exercise. Notably, a greater proportion of these individuals reported going out or exercising “almost every day,” whereas the low SRH group showed higher percentages of limited activity frequency (e.g., less than once per week). These differences were statistically significant (p < 0.001 for both dimensions), highlighting the multifaceted relationship between functional capacity, activity levels, and subjective health perceptions.

**Table 3:** Comparison of GDS, TMIG-IC, MFS, and activity frequency by SRH †Mann-Whitney U test (U value shown) ‡Chi-square test (χ² value shown) GDS, Geriatric Depression Scale; TMIG-IC, Tokyo Metropolitan Institute of Gerontology Index of Competence; MFS, Motor Fitness Scale; SRH, self-rated health; IQR, interquartile range

Variable	High SRH perception group (n = 1,337)	Low SRH perception group (n = 484)	Test statistic	P-value
GDS score (median, IQR)	0 (0-1)	2 (1-3)	U = 149,864	<0.001^†^
TMIG-IC score (median, IQR)	5 (4-5)	4 (2-5)	U = 399,626	<0.001^†^
MFS score (median, IQR)	13 (11-14)	9 (5-12)	U = 385,999	<0.001^†^
Mobility	6 (5-6)	3 (2-5)	U = 406,770	<0.001^†^
Strength	4 (4-4)	3 (1-4)	U = 398,554	<0.001^†^
Balance	4 (3-4)	2 (1-3)	U = 421,434	<0.001^†^
Frequency of going out (%)				
Less than once a week	28 (2.1)	40 (8.3)	χ^2^ = 83.302	<0.001^‡^
Once a week	65 (4.9)	47 (9.7)		
2-3 times a week	320 (23.9)	144 (29.8)		
4-5 times a week	392 (29.3)	119 (24.6)		
Almost every day	510 (38.1)	117 (24.2)		
No response	22 (1.6)	17 (3.5)		
Frequency of exercise (%)				
Less than once a week	88 (6.6)	75 (15.5)	χ^2^ = 65.398	<0.001^‡^
Once a week	102 (7.6)	58 (12.0)		
2-3 times a week	343 (25.7)	129 (26.7)		
4-5 times a week	310 (23.2)	91 (18.8)		
Almost every day	428 (32.0)	95 (19.6)		
No response	66 (4.9)	36 (7.4)		

Decision tree analysis

**Figure 1:**
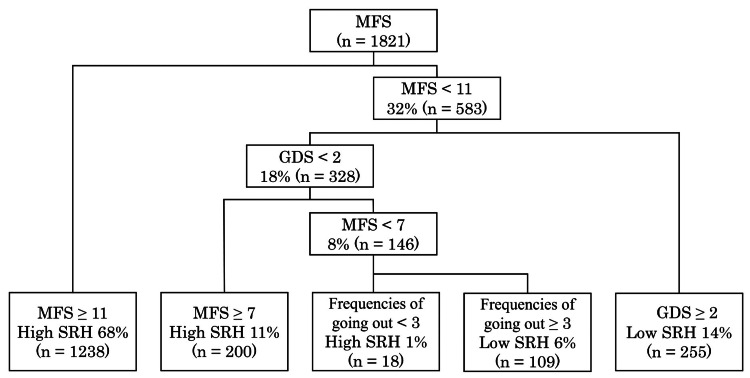
Classification tree identifying hierarchical determinants of SRH in community-dwelling older adults Classification tree identifying key determinants of self-rated health (SRH) among community-dwelling older adults. The model shows hierarchical splits by Motor Fitness Scale (MFS), Geriatric Depression Scale (GDS), and the frequency of going out. MFS ≥ 11 was the primary discriminator of high SRH, followed by GDS and going out frequency in lower-functioning subgroups

The CART analysis identified MFS as the most important determinant of SRH, with the initial split occurring at a score of 11. The participants with MFS scores of <11 were further stratified based on GDS, the frequency of going out, and other lifestyle factors. This cutoff point was data-driven and not based on any predefined clinical threshold; however, it may reflect a meaningful functional distinction in this population. Figure 1 illustrates the hierarchical structure of the decision tree model. Nodes predominantly predicting high SRH were characterized by higher motor fitness, lower depressive symptoms, and more frequent outdoor activities.

Variable importance

**Figure 2:**
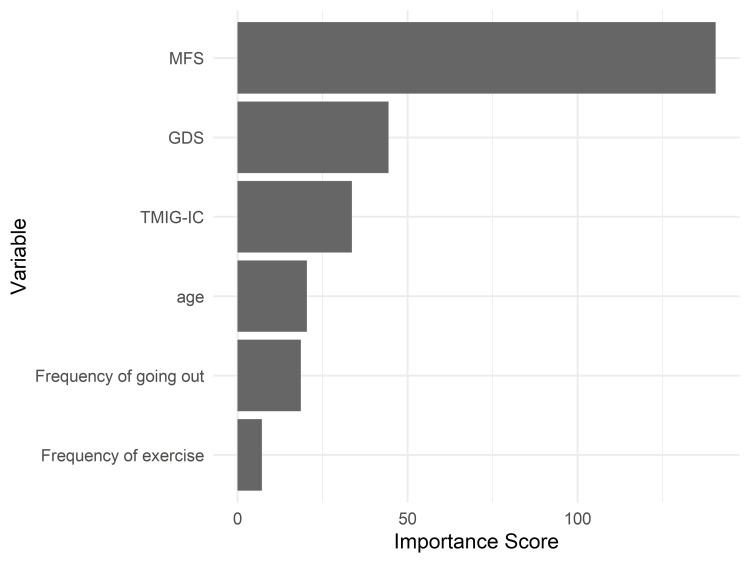
Variable importance in the decision tree model for predicting SRH Relative importance scores of predictors in the classification and regression tree (CART) model for self-rated health (SRH) classification. The Motor Fitness Scale (MFS) was the most influential variable, followed by the Geriatric Depression Scale (GDS), the Tokyo Metropolitan Institute of Gerontology Index of Competence (TMIG-IC), age, the frequency of going out, and the frequency of exercise. Scores reflect each variable’s contribution to reducing Gini impurity during tree construction

The relative importance of each predictor variable in the decision tree model was evaluated based on the cumulative reduction in the Gini impurity. As shown in Figure 2, MFS demonstrated the highest importance, indicating that it was the most influential variable for classifying SRH. This was followed by the GDS and the level of TMIG-IC. Other variables, such as age and the frequency of going out, contributed less to the classification. These results are consistent with the hierarchical structure observed in the decision tree and reinforce the central role of physical and mental health factors in shaping older adults’ subjective health perceptions.

Model performance

The CART model demonstrated satisfactory performance in classifying the participants based on SRH. The confusion matrix derived from this completed dataset showed an overall classification accuracy of 80.3%, with a sensitivity of 51.5% for identifying individuals with low SRH and a specificity of 90.8% for identifying those with high SRH (Table 4). Tenfold cross-validation yielded a mean classification accuracy of 78.9% (standard deviation: 2.0%), with fold-specific accuracies ranging from 76.6% to 82.4%, thereby demonstrating consistently stable performance across the imputed datasets.

**Table 4:** Confusion matrix for the classification of SRH using the decision tree model SRH: self-rated health

Actual/predicted	Predicted: high SRH	Predicted: low SRH	Total
Actual: high SRH	1,214	123	1,337
Actual: low SRH	235	249	497
Total	1,449	372	1,821

## Discussion

In this population-based study of community-dwelling older adults in Ayase City, Japan, we employed decision tree analysis to explore the hierarchical structure of factors associated with SRH. The model identified MFS as the most influential discriminator of SRH status, with an MFS score of 11 emerging as a critical threshold distinguishing those with high SRH status. Among individuals with lower MFS scores, depressive symptoms, as measured by the GDS, further stratified the SRH outcomes. Specifically, the participants with low MFS and high GDS scores (GDS ≥ 2, based on the decision tree split) were more likely to report low SRH. Additionally, among those with impaired motor fitness and minimal depressive symptoms, the frequency of going out played a nuanced but noteworthy role in differentiating perceived health status. Specifically, the model revealed that within this subgroup, individuals who went out more frequently (≥3 times per week) were more likely to report low SRH. This counterintuitive pattern suggests that frequent outings in this context may reflect obligation-driven activity, such as caregiving responsibilities or essential errands, rather than voluntary engagement, and may not correspond to a better perception of health. These findings underscore the layered interplay between physical function, mental health, and lifestyle factors in shaping subjective health perceptions among older adults [17-19].

The observed association between higher motor fitness and better SRH aligns with prior research linking physical performance to perceived health status in aging populations [20]. Functional mobility and balance, core components of MFS, are essential for maintaining independence and social engagement, both of which reinforce positive health perceptions [21]. Additionally, depressive symptoms, as measured by GDS, were a key secondary splitter in our decision tree, underscoring the psychological dimensions of SRH [22]. This supports existing evidence suggesting that psychological well-being significantly influences how older adults evaluate their overall health, often independent of clinical diagnoses [23].

Although TMIG-IC did not appear in the final decision tree structure, its relatively high variable importance score further illustrates the multidimensional nature of SRH [24]. Functional competence in daily life, such as managing finances or engaging in intellectual activities, likely reflects broader reserves of cognitive and physical health, contributing to a stronger sense of well-being [25]. Its absence from the tree may be explained by overlapping effects with MFS or GDS, which were selected earlier in the model. Alternatively, TMIG-IC may exert a more linear influence on SRH, making it less amenable to discrete split points used in decision tree algorithms.

The use of a decision tree model adds methodological value to the existing literature. Traditional multivariate regression models often assume linearity and may overlook complex interactions or threshold effects among variables. In contrast, decision trees allow for the intuitive visualization of conditional relationships and enable the identification of subgroups that may benefit from targeted interventions [26,27]. For instance, individuals with low motor fitness and elevated depressive symptoms may represent a particularly vulnerable subgroup requiring multifaceted support strategies [28]. Tailored interventions for this particularly vulnerable subgroup may involve combining structured physical activity with accessible mental health support. Such approaches align with broader community-based strategies discussed below.

Our model demonstrated satisfactory classification performance, with an accuracy of 80.3% and high specificity (90.8%) for detecting individuals with high SRH [7]. Although the sensitivity was more modest (51.5%), this trade-off reflects the model’s tendency to prioritize specificity, which is a common feature of tree-based algorithms [29]. Importantly, cross-validation confirmed the robustness of the model, supporting its generalizability to similar community settings [26]. From a practical perspective, the high specificity ensures reliable identification of individuals with high SRH, which may assist in prioritizing preventive and promotional health strategies for this group. However, the relatively low sensitivity indicates that a substantial portion of individuals with low SRH may not be identified by the model. This limitation is consistent with prior findings that decision tree models, while interpretable and accurate, may underperform in identifying minority classes [7,29]. Accordingly, this model should be complemented by additional screening approaches if the goal is to detect at-risk individuals with low subjective health perception.

From a public health perspective, the identification of motor fitness, depressive symptoms, and functional competence as key determinants of subjective health perception highlights the potential value of integrated, community-based interventions. For example, regular physical activity programs, neighborhood walking initiatives, and local social participation opportunities can help support both physical function and social engagement in older adults. In addition, accessible mental health services that offer depression screening and early support may address unrecognized psychological distress. Implementing such multifaceted and preventive strategies may contribute to promoting subjective health and overall well-being among aging populations.

Limitations

This study has several limitations. First, its cross-sectional design precludes any causal inference. Although the decision tree revealed meaningful associations and stratification patterns, it did not establish temporal relationships between the predictors and SRH outcomes. Compared with previous decision tree analyses of SRH conducted in rural China, our study similarly highlighted the importance of mental and physical health factors [10]. However, our model emphasized modifiable predictors and community-based applicability, offering a complementary perspective focused on intervention prioritization in urban Japanese settings. Second, despite the rigorous implementation of multiple imputation procedures, residual confounding and measurement errors may remain, particularly due to the self-reported nature of key variables such as SRH, GDS, and lifestyle indicators. Third, although the sample was relatively large and drawn from a community-dwelling population, it was limited to a single Japanese municipality, which may affect the generalizability of the findings to other populations and healthcare systems. However, Ayase City is a mid-sized suburban area with a demographic structure and aging rate that closely align with national averages. Therefore, while caution is warranted, the findings may be reasonably applicable to similar urban and suburban communities in Japan.

Fourth, although decision tree models offer high interpretability and clinical relevance, they can be sensitive to small variations in the data. Although overfitting was minimized through cross-validation and controlled splitting criteria, future research should consider complementary modeling techniques, such as random forests or gradient boosting machines, which may help improve the robustness and generalizability of the findings. Fifth, the relatively low sensitivity of the decision tree model may limit its utility in identifying individuals with low SRH. As such, it may be less suitable as a standalone screening tool and should ideally be complemented by other assessment strategies in public health settings. Finally, in line with the study’s focus on modifiable factors to enhance practical applicability, we intentionally excluded non-modifiable variables such as physician-diagnosed chronic conditions from the decision tree analysis. While this approach allowed us to prioritize intervention-relevant predictors, it may have reduced the comprehensiveness of the model by omitting well-established determinants of SRH. Future studies incorporating both modifiable and non-modifiable variables may offer a more holistic understanding of subjective health perception.

## Conclusions

Using a decision tree analytic approach, this study identified motor fitness, depressive symptoms, and IADL as the most influential, modifiable determinants of SRH among community-dwelling older adults in Japan. The application of the CART model enabled the visualization of hierarchical and non-linear interactions among physical, psychological, and lifestyle-related factors. Notably, the root node of the tree was based on motor fitness (MFS), suggesting that this easily measurable domain could serve as a practical first-line indicator in community-based health screening or assessment strategies. By deliberately excluding immutable variables such as educational background and physician-diagnosed conditions, the model prioritized factors amenable to intervention, thereby enhancing its real-world applicability.

These findings highlight the central role of physical performance and mental well-being in shaping subjective health perceptions. Community-based exercise programs to improve motor fitness and accessible mental health services offering depression screening and early intervention could be effective strategies to address these modifiable domains. The model’s capacity to stratify health status based on practical and addressable predictors underscores its potential utility in guiding individualized interventions and informing public health strategies. However, its modest sensitivity suggests that it may be less effective in identifying individuals with low SRH and should therefore be complemented by additional screening or assessment tools in such contexts. Future longitudinal studies are warranted to elucidate causal relationships and assess the effectiveness of targeted interventions derived from this model.
